# Impact of Paraben Exposure on Adiposity-Related Measures: An Updated Literature Review of Population-Based Studies

**DOI:** 10.3390/ijerph192316268

**Published:** 2022-12-05

**Authors:** Xinyun Xu, Haoying Wu, Paul D. Terry, Ling Zhao, Jiangang Chen

**Affiliations:** 1 Department of Nutrition, The University of Tennessee, Knoxville, TN 37996, USA; 2 Department of Medicine, Graduate School of Medicine, The University of Tennessee, Knoxville, TN 37920, USA; 3 Department of Public Health, The University of Tennessee, Knoxville, TN 37996, USA

**Keywords:** parabens, human exposure, adiposity-related measures (ARM), population-based studies, endocrine disruptors

## Abstract

Parabens are alkyl esters of p-hydroxybenzoic acid that are commonly used in pharmaceutical and cosmetic products. Humans are exposed to parabens when they use these products and through diet. There are growing concerns that paraben exposure can adversely impact human health. The endocrine-disrupting and obesogenic properties of parabens have been observed in animal studies and in vitro, prompting the increase in population-based studies of paraben exposure and adiposity-related endpoints. In this review, we summarize epidemiological studies published between 2017 and 2022 that examined paraben exposure in utero, between birth and adolescence, and in adulthood, in relation to adiposity-related measures. Overall, these studies provide some evidence that suggests that paraben exposure, especially during critical development windows, is associated with adiposity-related measures. However, we have noted several limitations in these studies, including the predominance of cross-sectional studies, inconsistent sample collection procedures, and small sample sizes, which should be addressed in future studies.

## 1. Introduction

Parabens are a series of alkyl esters of *p*-hydroxybenzoic acid. They are low-cost, broad-spectrum antimicrobial, and antiseptic preservatives with greater effectiveness against Gram-positive bacteria than Gram-negative bacteria. Therefore, parabens are widely used in cosmetics, personal-care products, and pharmaceuticals to suppress microbial growth and extend product shelf life [1,2,3]. Additional paraben exposure is linked to indoor air and dust, foodstuffs, and tap water [4]. Parabens have been detected in human serum, umbilical cord blood, urine, milk, and placental tissues, indicating systemic and early exposure to parabens in humans [5,6,7,8,9,10,11]. There is an appreciable correlation between the urinary paraben concentrations of pregnant women and their newborn infants, indicating the transfer of the compound from the mother to the fetus [12,13].

Parabens are endocrine disruptors with demonstrated estrogenic [14,15,16] and antiandrogenic effects [17,18]. Parabens also activate the thyroid hormone receptor [19], peroxisome proliferator-activated receptors (PPARs) [20,21], and glucocorticoid receptor (GR) [22,23]. Activation of PPARs and GR correlates with their adipogenic effects. Consistently, parabens have been shown to promote adipogenesis in cell models of mice and human origins [23]. Moreover, post-weaning exposure to methylparaben increases fat pad mass, whereas post-weaning exposure to either methylparaben or butylparaben suppresses serum markers of bone formation in chow-fed female C57BL/6J mice [24]. Furthermore, both parabens, butylparaben, in particular, modulate mesenchymal stem cell fates by promoting the adipocyte lineage at the expense of osteocytes and chondrocytes [20]. These in vitro and animal studies suggest the obesogenic potentials of parabens.

In this review, we summarized population-based studies published in the past five years to assess the association of paraben exposure with the risks of adiposity-related measures. Studies were obtained from searches of PubMed, using search terms such as “paraben,” “obesity,” and “BMI.” Studies were grouped according to the life stage of the study participants during exposure from in utero, birth through to adolescence, and adulthood. Studies that only focused on paraben biomonitoring using various biological matrices were not included in this review. In vivo and in vitro studies of parabens’ actions, the safety of parabens in cosmetics, and the role of parabens in reproductive systems and breast cancer have been reviewed elsewhere [25,26,27]; therefore, they are not in the scope of the current review.

## 2. Results and Discussion

### 2.1. Paraben Exposure in Utero and Adiposity-Related Measures

From 2017 to 2022, 13 studies explored the association between prenatal or in-utero paraben exposure and adiposity-related measures in children (Table 1). The majority (11 out of 13) of the studies were prospective in design. Biological matrices were collected at various gestational ages, with urine as the predominantly measured biological matrix. A few studies measured paraben concentrations using placentae or amniotic fluid [28,29,30,31]. The majority of these studies showed that parabens could cross the placental barrier [28,29,30,31]. Early life exposure to parabens is associated with increases in the gestational weight gain (GWG) rate [32], changes in height, head, hip, or arm circumference [28,30,33,34], z-scores of bodyweight [35], BMI or BMI z-scores [31,36,37], and overweight status in early childhood [38], although the direction of associations depends on the type of parabens and gender (Table 1). However, results from one large-scale (n = 1015 mother–child pairs) and one small-scale study (n = 99 mother–newborn pairs) did not show any clear association. In the larger study [39], most (>76%) of the children had already reached puberty at the time the adiposity-related measures were evaluated at age 11 [39]. Because the onset of puberty has an impact on body lean and fat mass [40], the dynamic and substantial increase in sex hormones in the participants could mask the impact of weak exogenous chemical exposures on body weight and fat mass. In the smaller study [29], only 20% of the amniotic fluid samples had detectable levels of methylparaben (MeP), and all other parabens were detected in less than 2% of the samples, reducing the statistical power of the analysis.

Overall, these 13 studies suffer from methodological issues that hamper the interpretation of the results. Most of the studies only collected single-spot samples. One major weakness of all the studies is the lack of information regarding whether urine samples were collected consistently in a defined manner or at a defined time point (for example, first-morning fasting urine), which would help ensure measurement validity and reliability. The variability in the timing of urine collection could have affected the exposure estimation for chemicals such as parabens with short half-lives [42]. Furthermore, with single-spot samples, the varied timing of exposure assessment in relation to gestational age may introduce exposure bias, in that the urinary paraben concentrations measured during the later gestational stage may not reflect the level of paraben exposure during the earlier pregnancy [43]. The standardization of exposure assessments should be, therefore, an important consideration in future studies. In addition, the concentrations of parabens in a sample depend on the source of paraben exposure and the time when the last exposure occurred before the sample collection [44]. When exposure to different parabens varies over time, repeated measures of exposure will reduce bias in exposure estimation. Finally, in all the studies that carried out postnatal follow-ups with the children, postnatal paraben exposure was not measured for these children; therefore, it is unknown how postnatal exposure of parabens might have contributed to the trajectories of adiposity measures in these studies.

Although the overall evidence supports an association between paraben exposure in utero and the risks of subsequent adiposity-related measures, we cannot make any conclusions regarding potential effect modifications by factors such as gender, which have shown conflicting results. Hu et al. reported that the adipogenic potency of parabens depends on the length of the linear alkyl chain [23]. Longer chain parabens are also more estrogenic compared to their shorter counterparts [45]. Estrogens play a critical role in the development of obesity [46,47] and parabens interfere with the estrogen metabolism pathway [48,49]. In vitro maternal butylparaben (BuP) exposure increases the weight of female offspring, but not male offspring mice [37], which may support the observed gender-specific effects in some of the studies, although the latter has not been consistent (Table 1) [30,33,34,35,36,37,41]. Both estrogen and androgen are critical in regulating adiposity and metabolism. The concentrations of endocrine disruptors in local tissues and organs may determine the unbalanced steroid hormone action [50]. Therefore, the exact molecular mechanisms responsible for the gender-specific effects of prenatal paraben exposure on several adiposity-related measures warrant further investigation.

It is also possible the different results from these studies are due to the heterogeneity in paraben exposure profiles in different populations [32]. Propylparaben (PrP) was the most relevant chemical in the mixture in Berger’s study conducted in the United States [38], while in the Spanish Environment and Childhood project (INMA), PrP minimally contributed to the mixture [39]. Paraben content can also vary substantially among different food products [51]. Furthermore, among the early-life adiposity-related measures (for example, gestational weight gain (GWG), GWG rates, length at birth, head, arm, hip, chest circumference, BMI, BMI z-score and percentage of fat), the most reliable predictor(s) for subsequent later-life health outcomes is yet to be determined [36,52,53,54,55].

The current literature suggests that prenatal exposure to parabens interferes with the programming of endocrine signaling pathways that could lead to the observed changes in adiposity in children. Therefore, as a precaution, pregnant women should consider reducing their use of cosmetic products with high paraben contents, using alternative products that do not contain parabens, and possibly reducing paraben exposure from dietary sources [51]. Future studies on prenatal paraben exposure and adiposity-related outcomes in children may need to quantify dietary paraben exposure and frequency of cosmetic product applications or the type of cosmetic products used before and during pregnancy in mothers, to improve the accuracy of exposure estimation. The collection of data from matrices, in addition to urine, should also be considered. For instance, as the largest fetal organ, the placenta may better reflect fetal paraben exposure [56]. Caution is also needed when interpreting data with a low detection rate of certain parabens in the biological samples used. In some studies, the concentrations of certain parabens in most samples were lower than the limit of detection (LOD); therefore, dichotomization (e.g., paraben levels were treated as a binary variable, either below LOD or above LOD) was used for data analysis [33,35,36], leading to the loss of information and statistical power [57]. Dichotomization may also increase the chance of false-positive findings [58]. In addition, the threshold effects and dose-dependent associations cannot be examined when dichotomization is applied [36]. A few studies used mixed-pollutant models to account for the complex exposure patterns of common endocrine disruptors while controlling for potential co-pollutant confounding [38,39]. However, models such as Bayesian hierarchical models (BMH) and Bayesian kernel machine regression (BKMR) cannot function with missing data and/or are sensitive to outliers [38,39,59], leading to reduced sample sizes. Future studies should continue to develop statistical models that more accurately and completely capture human chemical exposures.

### 2.2. Paraben Exposure during Adolescence and Adiposity-Related Measures

Six studies have explored the association between postnatal paraben exposure and adiposity-related measures in children of various ages (all under 20 years of age) (Table 2). Postnatally, pharmacological, personal-care products and dietary intake are the major sources of paraben exposure [60]. The majority (5 out of 6) of the studies were cross-sectional. Therefore, causal inference is limited, and the causal direction between these factors cannot be established. A one-time urine sample was the predominant matrix, although one study used daily intake of parabens from food sources to assess adiposity-related measures [41,51]. Results from these studies are mixed, showing positive [41,51,61], inverse [62,63], and null [64,65] associations. Further complicating the interpretations, one study found an association only in boys [61], whereas another found an association only in girls [51]. Thus, these studies do not clearly support any association between postnatal adolescent paraben exposure and adiposity-related measures. A major challenge with studies on adolescent exposure is the dynamic changes in sex hormones and the circadian rhythms of other hormones during the adolescent years, which are likely to affect paraben exposure. Standardized sample collection may help to minimize such confounding effects. In addition, these studies relied on a single-spot urine sample, which is another potential source of bias. Furthermore, most postnatal exposure studies did not collect dietary information [51,63]. If foods containing the most parabens are also the most calorific, then dietary confounding factors would present considerable threats to causal inference regarding paraben exposure [66].

Overall, the literature on paraben exposure and adiposity-related measures in adolescents remains sparse and inconsistent. As mentioned above, both methodologic and adolescent-specific sources of error hamper the interpretation of this literature. Prospective studies with standardized and repeated sample collection are needed. Studies should also capture dietary information to accurately estimate paraben exposure and identify confounding dietary factors, such as total energy intake.

### 2.3. Paraben Exposure during Adulthood and Adiposity-Related Measures

As in the case of adolescents, adults are also primarily exposed to parabens through pharmacological and personal-care products (PPCPs) and dietary sources [51]. Twelve studies in the past five years have investigated the association between the concentration of parabens in urine and adiposity-related measures in adults (Table 3). Overall, seven studies suggest an inverse association between paraben exposure and adiposity-related measures (Table 3). Urinary concentration of parabens has been shown to increase with the frequency of the use of personal-care products, specifically “leave-on products” or products applied over a wider skin surface [67]. Urinary concentration of parabens also varies with age, which may reflect age-related changes in lifestyle, food exposure, and endocrine disruptor metabolism rates [60]. As urine is the most common biological matrix in these studies, a major limitation is the lack of consistency in urine sample collection. In some studies, participants provided first-morning/early-morning urine samples [62,68,69], whereas, in other studies, the timing of urine collection was different, even within the same study [65,67,70]. In some studies, fasting morning samples were collected from at least a portion of the participants [7,67], while in other studies, no such information was provided [29,62,64,68,71,72,73]. Seasonal variability in EDS concentrations in urine, including parabens, is well documented [69,70]; however, seasonal timing of exposure was not considered in most of these studies.

BMI and/or waist circumference (predictors of abdominal adiposity) were measured in all twelve studies. Yet, the diet was not considered in most studies, which is an important source of paraben exposure and a confounder when adiposity-related measures are considered. The majority (10 of 12) of these studies were cross-sectional (Table 3); therefore, the results cannot be used to determine the causal relationship. Regarding prospective studies, one study had a small sample size (n = 73), with a single spot-urine sample collected nine years prior to the assessment of the adiposity-related measures [73]. Therefore, it is unknown whether the level of paraben exposure during this 9-year period remained constant or not. In another study, women in one group (the metabolic group) were approximately three years older than the non-metabolic group, but the participants’ menopausal status was not examined [73]. The menopausal transition period in aging women is strongly associated with weight gain [74].

One-third of the studies (4 of 12) did not find an association between parabens and BMI (Table 3), including the only study that considered dietary sources of parabens [7]. Moreover, these four studies were statistically underpowered, especially for sub-analysis by gender or other potential effect-modifying factors. Among the remaining eight studies, all but one [72] demonstrated an inverse relationship between paraben exposure and BMI, or that higher baseline paraben exposure was associated with reduced weight loss in response to a calorie-restriction intervention [75]. Interestingly, one of the largest studies, conducted in Korea with 3782 participants [72], showed that urinary ethylparaben (EtP) concentrations were positively associated with BMI, whereas a study conducted in the US using NHANES showed an inverse association [63]. The reasons for the inconsistency are unknown. However, the relatively short half-lives of parabens, varying uses of personal-care products by the two different populations [72], differences in the time frame during which the participants were recruited (2007–2014 in NHANES vs. 2015–2017 in Korean study) [63,72], the timing of sample collection, and unknown methodological issues or bias may have affected the estimation of urinary paraben concentrations, therefore influencing the strength and/or direction of the associations. For example, no fasting was required for those participants with afternoon or evening appointments to provide urine in the NHANES cohort (2007–2008) [76].

The association between urinary paraben concentrations and adiposity-related measures may also depend on how urinary paraben concentrations were adjusted. Urinary creatinine (UC), specific urinary gravity (SG), and covariate-adjusted standardization (CAS) are methods of adjustment for urine dilution, with UC and SG being the most common. The choice of the method should not be arbitrary; for example, in one study [72], a positive association between EtP and BMI was revealed when CAS was used to adjust paraben concentrations in urine but was not observed when UC or SG was used. The concentration of urinary creatinine is influenced by age, race/ethnicity, gender, and muscle mass. Future studies should determine if the use of the CAS adjustment method is valid across populations or race-/ethnicity-dependent. Therefore, the choice of urine normalization method should be validated and standardized.

## 3. Expert Opinion and Future Directions

Early-life determination of adult health theory suggests that there is a vulnerable biological window during which exposure to sufficient doses of endocrine disruptors is associated with an increased risk of adverse health outcomes [77]. Results of human studies from multiple countries indicate that parabens can cross the placental barrier [28,29,30,31]. Fetal exposure to parabens is associated with altered gestational weight gain [32], height, head, hip, or arm circumference [28,30,33,34,41], z-scores of weight and length [35], BMI z-scores [31,38], and overweight status in early childhood [38]. One of the most significant challenges in the interpretation of this literature is that few studies were initially designed to examine parabens. For instance, the environmental exposure components were often added at a later research stage, resulting in only one single-spot urine sample, or urine samples were missing for many study participants. Thus, estimates of paraben concentrations from many of these studies may not be representative of exposure over the targeted time periods. Several recent longitudinal cohort studies did not have an adequate sample size to detect small effects or allow stratified analyses based on gender, race/ethnicity, or pre-pregnancy BMI status [29,71,73]. Thereby, there is a great need to carry out large, long-term cohort studies with repeated measurements of chemical exposures across a broad range of developmental periods to elucidate their effects on childhood and adult health outcomes.

The absorption and metabolism of parabens depend on the length of the carbon chain, which varies across the types and brands of consumer products. The composition of paraben profiles in human samples is often population- and/or region-specific, influenced by the local environment, how the specific pharmaceutical and personal-care products are used, as well as by varying dietary cultures/lifestyles [51,68,72]. The existing literature suggests several other ways that future investigations may advance the current knowledge. For example, BMI or BMI-z scores may not be an appropriate outcome variable when measuring body development in newborns and preadolescents [53,54]. Instead, whole-body dual X-ray absorptiometry [36] might be more effective in estimating body composition in these age groups.

As in adolescence, pregnancy is a biological stage characterized by dynamic changes in circulating hormones, glucose, proteins, and kidney function, which could affect the osmolarity of urine samples [78,79]. Urinary analyte concentrations are also susceptible to variations by the time of the day [80] and the season when the samples are collected [69,70], inherited inter-individual differences in toxicokinetics, and physiological characteristics of the biomonitoring matrix [13]. Therefore, in addition to spot urine samples, these sources of variation need to be addressed when designing future studies. Urinary creatinine remains the most widely used method for adjusting urine dilution. Research from Lee’s group, however, showed that different methods of urine dilution adjustment might change the magnitude, and even the direction, of the associations between paraben exposure and metabolic-syndrome-related components [72]. Measurement of urinary analytes over 24 h is currently the most definitive method to quantify endocrine disruptor exposure. However, prolonged urine collection is inconvenient and often inaccurate due to frequent collection errors [81]. Alternatively, sampling biometrics, such as hair, may help to improve exposure assessment due to the accumulation of endocrine disruptors during hair growth. Hair is a relatively easy sample to obtain and analyze [82] and can, therefore, be sampled frequently, but the validity and repeatability of this method require further study.

Humans are exposed to multiple environmental pollutants daily. However, many published human studies reviewed here used single-pollutant models to assess paraben exposure and the associated health impacts. Bayesian mixture pollutant models might be a better analytical approach to account for complex exposure patterns and potential co-pollutant synergy [38,39]. Furthermore, many studies reported in this review measured exposure to parabens several years ago [7,32,33,36,39,63,64,67,70,72,73], which may not accurately reflect the types or levels of parabens prevalent in humans today. Paraben content in consumer products changes over time, partially due to the rise of public awareness and tightening regulatory guidelines in personal-care products and food [83,84]. Age, race, location, and gender-specific associations between paraben exposure and adiposity-related measures should be priorities of future studies due to the likelihood of higher endocrine disruptor exposure in vulnerable populations.

Finally, increasing evidence also highlights the importance of epigenetics as a functional modifier of the genome and a key determinant of disease risk [85]. For example, it has been suggested that the interaction of leptin receptor polymorphism and dietary intake of parabens may increase BMI [31]. Leptin receptors, together with leptin secreted from adipocytes, serve as a signal of satiety in the central nervous system, which controls food intake and subsequent energy expenditure. The inclusion of an epigenetic and/or genetic polymorphism component in future population studies may help to elucidate the interaction between paraben exposure and the key genes underlying obesity predisposition and outcomes [31,86,87].

## Figures and Tables

**Table 1:** Association of paraben exposure in utero and adiposity-related measures (ARM).

Study/Country	Sample Size	Year of Recruitment	Type of Study	Time of Sample Collection	Matrices	ARM	Major Shortcomings	Major Findings
Güil-Oumrait et al. 2022, Spain [39] ^a^	1015 mother–child pairs (500 males; 515 females)	2003–2008	Prospective	First and third trimesters	Urine	BMI; BMI z-score	High temporal variability in paraben concentrations due to the lack of serial urine collections over the pregnancy; health effects were assessed at asingle time point (11 years).	No association was identified between maternal urinary concentrations of parabens and adiposity measures of the child at 11 years of age.
Golestanzadeh et al. 2022, Iran [28] ^b^	128 pregnant women and 142 newborns	2019–2021	Cross-sectional	During C-section	Amniotic fluid	Newborn weight, head, chest, hip, and arm circumference	Only women who underwent C-sections were enrolled (selection bias); small sample size; only one spot sample was collected; cross-sectional nature of the study design; no maternal caloric intake was considered in the analysis.	BuP concentrations in the amniotic fluids were positively associated with weight, hip, and arm circumference but negatively correlated with height, head, and chest circumference of the newborns. MeP concentrations in the amniotic fluids were negatively associated with head circumference, chest, hip, and arm circumference but positively associated with the height of the newborns; EtP concentrations in the amniotic fluids were negatively associated with arm circumference but positively associated with the height of the newborns; PrP concentrations in the amniotic fluids were negatively associated with arm circumference of the newborns.
Reimann et al. 2021, Belgium [31] ^c^	218 mother–child pairs, (112 males and 106 females)	2014–2017	Prospective	During delivery	Placenta	BMI z-score	No information was available for the child’s breastfeeding status, caloric intake, or postnatal paraben exposure; only one spot sample was collected.	Placental EtP concentrations were negatively associated with children’s BMI z-scores.
Hojsager et al. 2021, Demark [36] ^d^	312 mother–child pairs	2010 and 2012	Prospective	Early third trimester	Urine	Fat mass, body mass; BMI z-score	The women enrolled in the study were older and more often nulliparous compared to the background population (selection bias); no information was available about weight gain during pregnancy; only one spot sample was collected; childhood exposure to parabens was not assessed; childhood caloric intake was not considered in the analysis.	Maternal urinary BuP concentrations were positively associated with total body fat percentage and android fat percentage in boys.
Karzi et al. 2021, Greece [29]	99 mother–newborn pairs (41 males; 44 females)	Unknown	Prospective	Second trimester	Urine/amniotic fluid	Birth weight, length, and head circumference	Low prevalence of parabens detected in amniotic fluid; small sample size; only one spot sample was collected; maternal caloric intake was not considered in the analysis.	No association was identified between maternal parabens in either urine or amniotic fluid and adiposity-related measures in the newborns.
Berger et al. 2021, United States [38] ^e^	309 mother–child pairs	Since 1999	Prospective	First and second trimesters	Urine	BMI, BMI z-score; overweight/obese status	Lack of ability to assess sex-specific associations; childhood exposure to parabens was not assessed.	Maternal urinary PrP concentrations were positively associated with BMI z-scores and overweight/obesity status of the child at 5 years of age.
Vrijens et al. 2020, Belgium [30]	142 mother–child pairs (74 males; 66 females)	2014–2016	Cross-sectional	During delivery	Placenta	Birth weight, length, and head circumference	Cross-sectional nature of the study design; small sample size; only one spot sample was collected; maternal caloric intake was not considered in the analysis.	Placental total parabens were negatively associated with birth weight and head circumference in girls; EtP concentrations were negatively associated with head circumference in girls.
Wen, et al. 2020, China [32]	613 pregnant women	2014–2015	Prospective	During each trimester	Urine	Gestational weight gain (GWG) and GWG rate (kg/week)	Neither the frequency of use of personal care products during pregnancy nor maternal caloric intake was considered in the analysis.	First-trimester MeP, EtP, PrP, and total parabens levels in the women’s urine were positively associated with an increase in the GWG rate of the first-trimester, and these associations were stronger than those of the second or third trimesters.
Jamal et al. 2020, Iran [33]	189 mother–child pairs (66 males; 92 females)	2016	Prospective	First trimester	Urine	Birth weight, length, and head circumference	Small sample size; only one spot sample was collected; maternal caloric intake was not considered in the analysis.	Maternal urinary BuP concentrations were positively associated with the birth weight of boys; maternal urinary PrP concentrations were negatively associated with the birth length of girls; maternal urinary MeP and BuP concentrations were positively associated with the head circumference in girls.
Leppert, et al. 2020, German [37] ^f^	626 mother–child pairs, (108 males; 115 females)	2006–2008	Prospective	Third trimester	Urine	BMI	No information was available for postnatal paraben exposure; only one spot sample was collected; childhood caloric intake was not considered in the analysis.	Maternal urinary BuP concentrations were positively associated with the overweight status of the children within the first eight years of life, with a stronger trend observed in girls.
Chang et al. 2019, Taiwan [34]	199 mother–child pairs (99 males; 100 females)	2014–2015	Prospective	Third trimester	Urine	Birth weight, body length, head, and thoracic circumference; Ponderal Index	Small sample size; highly educated pregnant women were enrolled in the study (selection bias); only one spot sample was collected; maternal caloric intake was not considered in the analysis.	Maternal urinary MeP concentrations were positively associated with the head circumference and Ponderal Index in boys; maternal urinary MeP concentrations were negatively associated with the birth weight, length, head circumference, and thoracic circumference in girls.
Wu, et al. 2019, China [35] ^g^	850 mother–child pairs (446 males; 404 females)	2014–2015	Prospective	During each trimester	Urine	Z-scores for weight and height	High temporal variability in paraben concentrations throughout pregnancy; postnatal paraben exposure was assessed.	Maternal urinary EtP concentrations were negatively associated with the weight z-scores of the child at birth; third-trimester urinary EtP concentrations were negatively associated with the weight z-scores at birth, 1 and 2 years in boys.
Wu, et al. 2017, China [41]	1016 mother–child pairs (527 male; 489 females)	2012–2014	Prospective	Within three days before delivery	Urine	Birth length and weight	Only one spot sample was collected; maternal caloric intake was not considered in the analysis.	Maternal urinary MeP concentrations were positively associated with the birth length in boys.

Footnotes: ^a^ BMI information was collected at 11 years of age; ^b^ number of male and female newborns was not provided; ^c^ a subset of 63 newborns was followed for 29 months to monitor BMI z-scores; ^d^ number of male and female newborns was not provided; adiposity-related measures were assessed at 7 years of age; ^e^ number of male and female newborns was not provided; adiposity-related measures were assessed at 5 years of age; ^f^ newborns were followed from 1 to 8 years of age; ^g^ adiposity-related measures were assessed at birth, 6 months, 1, and 2 years of age.

**Table 2:** Association of paraben exposure during adolescence and adiposity-related measures (ARM).

Study/Country	Sample Size (Male/Female)	Year of Recruitment	Type of Study	Matrices	Time of ARM Assessment (Years of Age)	ARM	Major Shortcomings	Major Findings
Monteagudo et al. 2021, Spain [51]	585 (313/272)	2017–2018	Cross-sectional	Food	12–16	BMI	Only the dietary sources of paraben exposure were assessed; cross-sectional nature of the study design; paraben concentrations were not measured in biological samples; caloric intake was not considered in the analysis.	High total parabens and MeP daily intake were associated with high BMI in girls but not boys.
Feizabadi et al. 2020, Iran [62]	100 (50/50)	unknown	Cross-sectional	Urine	12–20	Weight, BMI; waist circumference	Small sample size; cross-sectional nature of the study design; only one spot sample was collected; caloric intake was not considered in the analysis.	Inverse associations were identified between urinary concentrations of paraben (MeP and EtP) and BMI.
Kim et al. 2020, Canada [64]	1418 (695/723)	2014–2015	Cross-sectional	Urine	3–17	Height, weight, BMI; waist circumference	Cross-sectional nature of the study design; only one spot sample was collected.	No association was identified between urinary paraben concentrations and BMI z-score or waist circumference.
Quirós-Alcalá et al. 2018, United States [63]	1324 (684/640)	2007–2012	Cross-sectional	Urine	6–19	BMI z-score; waist circumference	Cross-sectional nature of study design; only one spot sample was collected.	Negative associations were identified between urinary MeP, PrP, and total parabens concentrations and the prevalence odds ratios of being obese vs. normal weight; negative associations were identified between urinary MeP, PrP, and total parabens concentra-tions and waist circumference. The associations were stronger in girls.
Deierlein et al. 2017, United States [65] ^a^	1017 (0/1017)	2004–2007	Prospective	Urine	12.8–18.4	Weight, height, waist circumference, BMI; percent body fat	Only females were enrolled in the study; only one spot sample was collected; caloric intake was not considered in the analysis.	No association was identified between baseline total paraben concentrations in urine and girls’ adiposity-related measures.
Guo et al. 2017, China [61]	436 (221/215)	2012–2013	Cross-sectional	Urine	3	Weight z-score, height z-score, weight for height z- score; BMI z-score	Cross-sectional nature of study design; only one spot sample was collected; caloric intake was not considered in the analysis.	Urinary EtP concentrations were positively associated with weight z-scores and height z-scores. Total paraben concentrations were associated with anthropometric measures only in boys.

Footnotes: ^a^ Girls were enrolled at 6–8 years of age. Adiposity-related measures were assessed yearly or biannually until they were 15.6 years of age on average (range: 12.8–18.4 years of age); weight, height, and waist circumference were recorded at baseline. BMI, waist circumference, and percentage of fat were measured at each visit.

**Table 3:** Association of paraben exposure during adulthood and adiposity-related measures (ARM).

Study/Country	Sample Size (Male/Female)	Year of Recruitment	Type of Study	Matrices	Time of ARM Assessment (Years of Age)	ARM	Major Shortcomings	Major Findings
Jala et al. 2022, India [71]	52 (0/52)	2020	Cross-sectional	Urine	18–31	BMI; waist-to-hip ratio (WHR)	Small sample size; cross-sectional nature of the study design; only females were enrolled in the study (selection bias); only one spot sample was collected; caloric intake was not considered in the analysis.	No association was identified between urinary paraben concentrations and BMI or waist-to-hip ratio.
Vindenes et al. 2021, Norway [67]	496 (258/238)	2014–2015	Cross-sectional	Urine	18.1 to 47.5	BMI	Cross-sectional nature of the study design; only one spot sample was collected; caloric intake was not considered in the analysis.	Urinary concentrations of MeP and EtP were negatively associated with BMI.
van der Meer et al. 2021, the Netherlands [75] ^a^	218 (70/148)	2008–2010	Prospective intervention	Urine	Average 52	BMI, waist circumference, body fat percentage	The study did not have a control group.	Higher baseline urinary paraben exposures were associated with reduced weight loss in a calorie-restriction intervention.
Lee et al. 2021, Korea [72]	3782 (1648/2134)	2015–2017	Cross-sectional	Urine	19–86	BMI	Cross-sectional nature of the study design; only one spot sample was collected; caloric intake was not considered in the analysis.	Urinary EtP concentrations were positively associated with BMI.
Zamora et al. 2021, Mexico [73]	73(0/73)	2008	Prospective	Urine	46.6 ± 6.3	BMI; waist circumference	Small sample size; potential confounding effect caused by the menopausal status of the participants; only females were enrolled (selection bias); only one spot sample was collected; paraben exposure was assessed 9 years prior to the measurement of adiposity-related outcomes; caloric intake was not considered in the analysis.	No association was identified between urinary paraben concentrations and BMI or abdominal obesity.
Karzi et al. 2021, Greece [29] ^b^	99 (0/99)	Unknown	Cross-sectional	Urine	18.0 to 44.0	BMI	Small sample size; cross-sectional nature of the study design; only females were enrolled (selection bias); only one spot sample was collected; caloric intake was not considered in the analysis.	No association was identified between urinary paraben concentrations and BMI.
Kim et al. 2020, Canada [64]	1137 (568/569)	2014–2015	Cross-sectional	Urine	46.2 ± 0.3	BMI; waist circumference	Cross-sectional nature of the study design; only one spot sample was collected.	Negative associations were identified between urinary MeP and total parabens concentrations and obesity and waist circumference in women.
Kiani Feizabadi et al. 2020, Iran [68]	178 (75/103)	Unknown	Cross-sectional	Urine	43.7 ± 11.8	BMI	Small sample size; cross-sectional nature of the study design; only one spot sample was collected; caloric intake was not considered in the analysis.	Negative associations were identified between urinary MeP concentrations and BMI.
Hajizadeh et al. 2020, Iran [69] ^c^	95 (0/95)	2018	Cross-sectional	Urine	34.2 ± 8.2	BMI; waist circumference	Small sample size; cross-sectional nature of the study design; only females were enrolled (selection bias); only one spot sample was collected; caloric intake was not considered in the analysis.	Negative associations were identified between urinary EtP concentrations and BMI.
Bethea et al. 2020, United States [70] ^d^	766 (0/766)	2010–2012	Cross-sectional	Urine	23–34	BMI	Participants were recruited from a single urban area (selection bias); cross-sectional nature of the study design; only one spot sample was collected; no dietary information was available.	Urinary concentrations of MeP and BuP were negatively associated with morbid obesity (BMI ≥ 35 kg/m^2^) compared to BMI < 25.
Yu et al. 2019, China [7]	562 (550/12)	2013–2015	Cross-sectional	Urine	22–59	BMI	Cross-sectional nature of the study design; only one spot sample was collected; only 12 women were enrolled (unbalanced study design); caloric intake was not considered in the analysis.	No correlations were identified between urinary concentrations of parabens (MP, EtP, and PrP) and BMI.
Quirós-Alcalá et al. 2017, United States [63]	4730 (2306/2424)	2007–2014	Cross-sectional	Urine	49.6 ± 17.4	BMI; waist circumference	Only one spot sample was collected; cross-sectional nature of the study design.	Urinary MeP concentrations were negatively associated with prevalence odds ratios for obesity and adiposity measures; Stronger associations were observed in females.

Footnotes: ^a^ All participants with a BMI > = 27 kg/m^2^ were enrolled; ^b,c^ only pregnant women were enrolled in these studies; ^d^ a study of a vulnerable population from Detroit, Michigan metropolitan area.

## Data Availability

Not applicable.
